# A Rare Case of Captopril-Induced Parotid Gland Enlargement

**DOI:** 10.7759/cureus.34339

**Published:** 2023-01-29

**Authors:** Ahmed Khan, Malak Alshehri, Ahad Babkier, Kutaiba Alahmad, Wisam Siam

**Affiliations:** 1 Department of Anesthesia, Al Noor Specialist Hospital, Makkah, SAU; 2 Department of Medicine and Surgery, Umm Al-Qura University, Makkah, SAU; 3 Department of Medicine, Ibn Sina National College for Medical Studies, Jeddah, SAU; 4 Emergency Department, Al Noor Specialist Hospital, Makkah, SAU

**Keywords:** adverse drug events, rare case, parotid enlargement, captopril, case report

## Abstract

The use of captopril has been linked to scarce adverse events characterized by parotid glands enlargement. We report a case of captopril-induced parotid enlargement in a patient with uncontrolled hypertension. A 57-year-old male presented to the emergency department (ED) with an acute state of headache. The patient has a background of untreated hypertension for which he was managed in the emergency department (ED) by the administration of captopril 12.5 mg sublingually to control his blood pressure. Shortly after the drug administration, he started to experience bilateral painless enlargement of parotid glands that resolved a few hours after the drug was withdrawn.

## Introduction

Captopril is an angiotensin-converting enzyme (ACE) inhibitor, a pharmaceutical medication approved by the Food and Drug Administration (FDA), to treat hypertension and left ventricular dysfunction with or without myocardial infarction [1,2]. In addition, it is important to note that ACE is used to treat diabetic nephropathy as they protect against diabetic and non-diabetic renal damage. Nevertheless, the mechanism is not fully understood [1,2].

Captopril has various adverse effects that are generally well tolerated, including skin rashes, changes in taste, and dry cough [3]. However, serious side effects have also been reported, including neutropenia, anemia, thrombocytopenia, hypotension, proteinuria, abnormal liver function tests, and stomatitis [3]. Rare adverse events have also been reported, including angioedema [4,5].

## Case presentation

**Figure 1:**
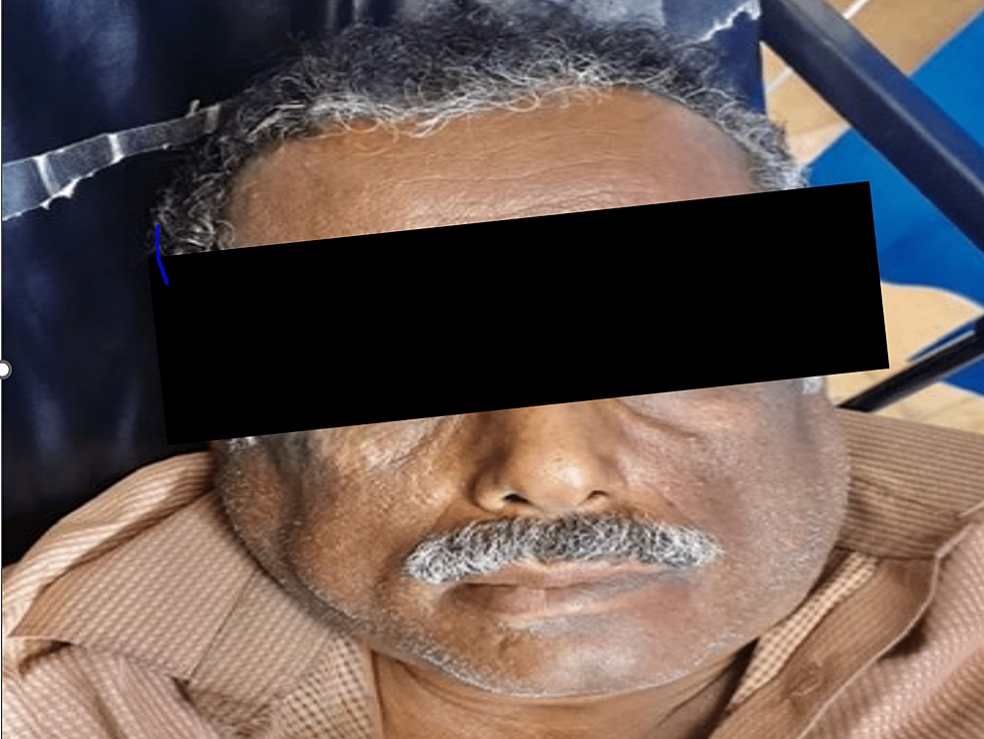
Bilateral parotid glands enlargement

**Figure 2:**
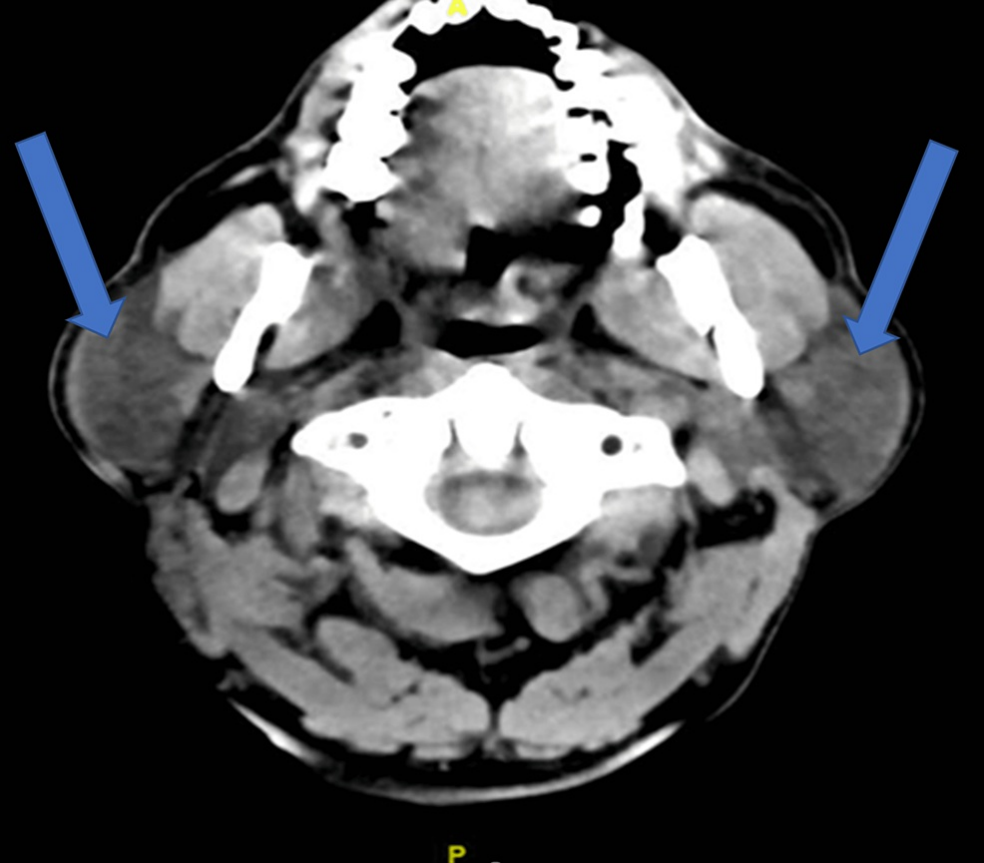
Brain CT showing bilateral parotid gland enlargement

A 57-year-old male patient presented to the emergency department (ED) in our facility complaining of a headache associated with slurred speech and swallowing difficulty that had started one day previously. The patient denied any history of head trauma, visual disturbance, paraesthesia, dysphasia, neck stiffness, fever, or dizziness. His medical history was significant for uncontrolled hypertension not on any medication. He had experienced no similar symptoms previously. Upon examination, he was afebrile, Glasgow Coma Scale (GCS) 14/15. His vital signs revealed a normal pulse of 85 beats per minute, oxygen saturation of 99% on room air, and blood pressure of 183/116 mmHg, which was diagnosed as a hypertension emergency. Neurological examination was significant for left-sided weakness and mouth deviation. Muscle tone and reflexes were normal, power of 4/5, with no abnormal movement, muscle wasting, fasciculations, spasticity, or rigidity. Cranial nerve function, sensation, and coordination were intact. The physical examination was otherwise unremarkable. Initial laboratory workups including complete blood count (CBC) and blood chemistry were within normal range. Brain computed tomography (CT) revealed a suspicious focal hypodensity of the external capsule and right parietal lobe with a suspicious area of loss of gray-white matter differentiation of the right insula likely to represent a recent infarction. Thus, initial management was started using captopril sublingual 12.5 mg to control his blood pressure and avoid a harmful decrease in cerebral perfusion as the patient was confirmed to have brain infarction. The aim was to reduce blood pressure by 20%-25% within the first four hours. However, after 40 minutes of drug administration, the patient started to experience bilateral painless progressive parotid gland enlargement (Figure 1). However, while the brain CT was obtained to confirm the diagnosis, it showed the presence of parotid enlargement bilaterally (Figure 2).

**Figure 3:**
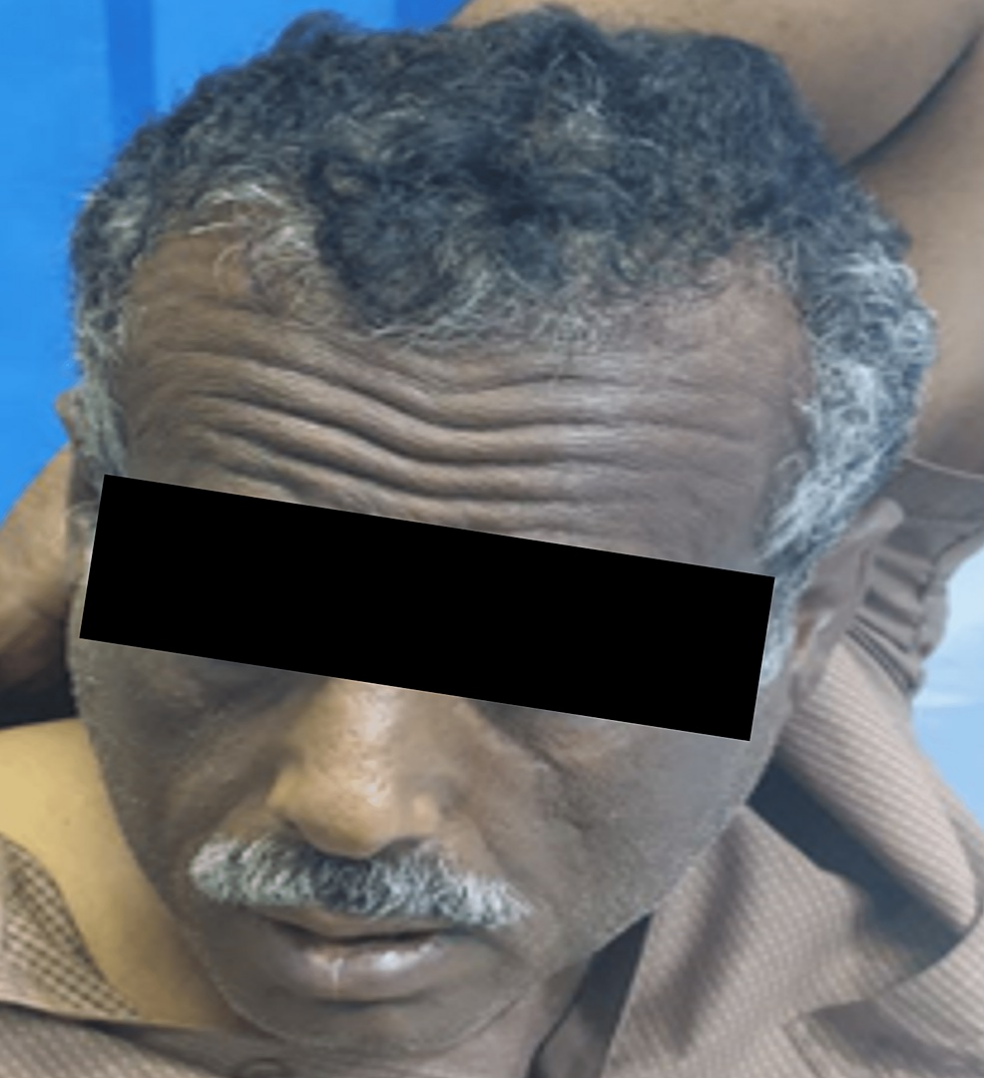
After four hours of drug withdrawal

Numerous factors may contribute to the acute enlargement of parotid glands. However, ACE inhibitor-induced angioedema was ruled out as the patient showed no signs of difficulty in breathing, eye or lip swelling, urticarial eruption, hives, wheezing, or loss of consciousness. The absence of pain, prodromal symptoms, purulent discharge, and the patient's initial laboratory investigations ruled out viral and bacterial infections in this patient (Table 1). Moreover, his past medical history was not significant for trauma or medication use. Therefore, captopril was recognized as the most likely cause of bilateral parotid enlargement. The patient was kept under observation, and four hours after drug wearing off, the swelling gradually disappeared as shown in Figure 3.

**Table 1:** Patient's initial laboratory investigations CK: creatine kinase, CPK: creatine phosphokinase.

Test	Result	Reference range
White blood cell (WBC)	8.12 (×10^9/L)	4-11
Red blood cell (RBC)	4.06 (×10^12/L)	4.5-5.5
Hemoglobin (HGB)	128 (g/L)	130-170
Hematocrit (HCT)	0.37 (L/L)	0.4-0.5
Mean corpuscular volume (MCV)	91.4 (fL)	83-101
Mean corpuscular hemoglobin (MCH)	31.5 (Pg)	27-32
Mean corpuscular hemoglobin concentration (MCHC)	345 (g/l)	315-345
Platelets	124 (×10^9/L)	150-400
Blood urea nitrogen (BUN)	3.54 mmol/L	2.6-6.4
Creatinine	82 µmol/L	62-115
Calcium	2.1 mmol/L	2.1-2.55
Sodium	142 mmol/L	135-145
Albumin	26 g/L	34-50
Potassium	3.7 mmol/L	3.5-5.1
CK/CPK	116 (U/L)	39-308

## Discussion

Drug-induced parotid enlargement is considered a scarce adverse event [6]. Although bilateral parotid enlargement is less prevalent than unilateral enlargement, the etiology of this condition is probably multifactorial. The primary contributor is a decrease in parotid salivary volume and flow rate. Further potential causes of decreased flow include stones, strictures, external duct pressure, mucus plugs, and congenital anomalies [7].

Despite the existence of several cases, drug-induced parotid enlargement remains a rare condition with an unclear mechanism [8]. To the best of our knowledge, this is the first case report of captopril-induced parotid enlargement in Saudi Arabia. Likewise, in 2016, a case was reported of a patient with end-stage renal disease who developed bilateral painless parotid enlargement three hours after captopril administration [8]. Along the same lines, in 2004, two cases were reported of the captopril-induced bilateral parotid and submandibular sialadenitis after one hour of drug administration. Neither patient had previous exposure to captopril [3]. 

The optimal management for this condition is drug elimination, as it has been reported that parotitis subsides within a few hours following treatment discontinuation [8]. The drug elimination duration varies according to the severity and the patient’s medical background. In our case, the parotid gland size was reduced four hours after discontinuation of captopril. Similarly, two patients with bilateral parotid and submandibular sialadenitis exhibited clinical improvement after four and 12 hours, respectively [3]. On the other hand, Mahdiabadi and Nikvarz reported that salivary gland enlargement nearly resolved after five days in a patient with end-stage renal disease (ESRD); this prolonged recovery period was attributed to renal failure, which increases captopril's elimination half-life [8]. The rapid improvement in the patient's condition compared to the few cases reported in the literature highlights this as a unique case.

## Conclusions

A substantial correlation was observed between the parotid enlargement reported in this case and the administration of captopril, constituting a drug-related adverse event. Captopril should be considered a possible cause of the acute parotid enlargement, despite the rarity of the adverse events associated with this medication. Regular monitoring of the patient's condition could be beneficial in determining the onset and excluding other etiologies. To our knowledge, this is the first case reported in Saudi Arabia.
